# metaboprep: an R package for preanalysis data description and processing

**DOI:** 10.1093/bioinformatics/btac059

**Published:** 2022-02-04

**Authors:** David A Hughes, Kurt Taylor, Nancy McBride, Matthew A Lee, Dan Mason, Deborah A Lawlor, Nicholas J Timpson, Laura J Corbin

**Affiliations:** MRC Integrative Epidemiology Unit at the University of Bristol, Bristol BS8 1TH, UK; Population Health Science, Bristol Medical School, University of Bristol, Bristol BS8 1TH, UK; MRC Integrative Epidemiology Unit at the University of Bristol, Bristol BS8 1TH, UK; Population Health Science, Bristol Medical School, University of Bristol, Bristol BS8 1TH, UK; MRC Integrative Epidemiology Unit at the University of Bristol, Bristol BS8 1TH, UK; Population Health Science, Bristol Medical School, University of Bristol, Bristol BS8 1TH, UK; NIHR Bristol Biomedical Research Centre, University of Bristol, Bristol BS8 1TH, UK; MRC Integrative Epidemiology Unit at the University of Bristol, Bristol BS8 1TH, UK; Population Health Science, Bristol Medical School, University of Bristol, Bristol BS8 1TH, UK; Bradford Institute for Health Research, Bradford Teaching Hospitals NHS Foundation Trust, Bradford BD9 6RJ, UK; MRC Integrative Epidemiology Unit at the University of Bristol, Bristol BS8 1TH, UK; Population Health Science, Bristol Medical School, University of Bristol, Bristol BS8 1TH, UK; NIHR Bristol Biomedical Research Centre, University of Bristol, Bristol BS8 1TH, UK; MRC Integrative Epidemiology Unit at the University of Bristol, Bristol BS8 1TH, UK; Population Health Science, Bristol Medical School, University of Bristol, Bristol BS8 1TH, UK; MRC Integrative Epidemiology Unit at the University of Bristol, Bristol BS8 1TH, UK; Population Health Science, Bristol Medical School, University of Bristol, Bristol BS8 1TH, UK

## Abstract

**Motivation:**

Metabolomics is an increasingly common part of health research and there is need for preanalytical data processing. Researchers typically need to characterize the data and to exclude errors within the context of the intended analysis. Whilst some preprocessing steps are common, there is currently a lack of standardization and reporting transparency for these procedures.

**Results:**

Here, we introduce *metaboprep*, a standardized data processing workflow to extract and characterize high quality metabolomics datasets. The package extracts data from preformed worksheets, provides summary statistics and enables the user to select samples and metabolites for their analysis based on a set of quality metrics. A report summarizing quality metrics and the influence of available batch variables on the data are generated for the purpose of open disclosure. Where possible, we provide users flexibility in defining their own selection thresholds.

**Availability and implementation:**

*metaboprep* is an open-source R package available at https://github.com/MRCIEU/metaboprep.

**Supplementary information:**

Supplementary data are available at *Bioinformatics* online.

## 1 Introduction

In the last decade, the study of chemical products arising from biological processes has moved from chemometrics to epidemiology (Ala-Korpela, 2015). In particular, the use of metabolomics as a functional read-out of an individual’s current health is becoming increasingly popular (Miggiels *et al.*, 2019). With rapid advances in technology and bioinformatics enabling the quantification of hundreds or even thousands of metabolites from a single biological sample, there is potential for these measurements to reveal valuable insights into biology and health. Both mass spectrometry (MS) and nuclear magnetic resonance (NMR) are common technologies used in these untargeted studies. Typically, laboratories have their own established protocols in sample preparation, generation of standards and controls and corrections for instrument and run day variability. Work is also underway to develop a common set of best minimum practices and reporting standards for laboratories to apply to ensure metabolomics data generation is robust and to enable harmonization across laboratories (Beger *et al.*, 2019; Evans *et al.*, 2020). As a result, researchers are now able to access high quality curated metabolomics data at scale.

After data generation by core facilities and prior to statistical analysis, researchers perform a series of data characterization and preanalytical preparation steps. These may include (i) the identification of samples of poor quality, (ii) the identification of metabolites that have unfavorable statistical properties and/or may not provide sufficient data for study analyses and (iii) the characterization of statistical properties of the data that may be relevant to downstream analyses. The latter is needed to help inform decisions involving data normalizations, transformations and analytical considerations that revolve around missing data.

Based on both our own experience and emerging literature published in this area (Barnes, 2020; Monnerie *et al.*, 2020), it is clear that approaches for post-acquisition, preanalytical data processing are varied both within and across analytical platforms. The general lack of methodological standardization makes combining and comparing data and results across studies difficult, thus impairing cross-study inference. Into this context, papers and researchers have recently called for standardization and transparency in reporting of metabolomic studies (Begou *et al.*, 2018; Karaman, 2017; Long *et al.*, 2020; Playdon *et al.*, 2019; van Roekel *et al.*, 2019). To some extent, this situation mirrors that seen in the field of genomics a decade ago; here, researchers responded with the development of standard protocols supported by open-source software tools such as EasyQC (Winkler *et al.*, 2014). Under the assumption that, as in genomics, collaboration and independent replication will be key in the utilization of metabolomics data going forward, it is important that this field progresses in optimizing workflows and recognizing where consistency can be achieved. In addition, whilst useful contributions have been made to facilitate improved transparency in the reporting of preanalytical data processing (Considine and Salek, 2019), it is clear that much needs to be done in this area (Considine *et al.*, 2018).

This paper introduces *metaboprep*, an R package developed to help those working with curated metabolomics data to achieve transparent and informed processing of their study sample data prior to statistical analysis. The package provides a detailed summary of the data, highlighting properties relevant both to setting sample/metabolite filtering criteria and to downstream analytical choices. *Metaboprep* can process any flat text data file containing curated metabolomics data with minimal formatting. In addition, the *metaboprep* package is currently able to process data as supplied by two of the main biotech companies operating in this sector—^1^H-NMR data from Nightingale Health^©^ (Helsinki, Finland) and ultrahigh-performance liquid chromatography–tandem mass spectrometry (UPLC-MS/MS) data from Metabolon (Research Triangle Park, NC, USA). We demonstrate the use of *metaboprep* using the Born in Bradford (BiB) cohort, including 1000 pregnant women with UPLC-MS/MS data (Metabolon), and The Avon Longitudinal Study of Parents and Children (ALSPAC), a birth cohort with 3361 samples collected during early adulthood and analyzed by NMR (Nightingale Health).

## 2 Materials and methods

### 2.1 Overview

*Metaboprep* is an R package designed to standardize the steps involved in preparing population level metabolomics datasets for statistical analysis. It was written using R (version 3.6.0) (R Core Team, 2019), is dependent upon R version 3.4.0 or greater and is available on GitHub (https://github.com/MRCIEU/metaboprep). A README is available on the *metaboprep* GitHub page that provides detailed instructions for running the *metaboprep* pipeline, as well as a Wiki page with details of the pipeline itself (Supplementary Data S1). All analyses performed in this manuscript used R (version ≥ 3.4.0); code is available on the repository providing a walk-through of the *metaboprep* package. There is also an example dataset provided on the GitHub repository for users to test and explore the utility of *metaboprep*. Data used here are available upon application from BiB and ALSPAC.

### 2.2 Data processing and filtering pipeline

**Fig. 1.**
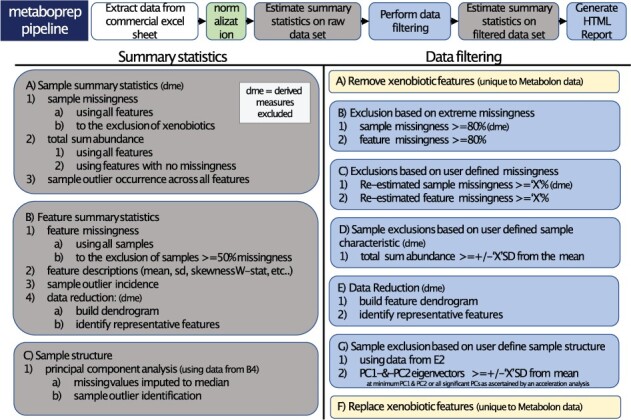
Brief description of the *metaboprep* pipeline. Along the top, the six primary steps the pipeline takes are outlined. The column on left provides an outline of the steps for the generation of summary statistics whilst the right provides an outline of the steps taken for sample and metabolite filtering. Common abbreviations used are: ‘dme’ for derived measures excluded; SD for standard deviations; ‘X’ which denotes a threshold variable that is defined by the user in the pipeline parameter file; PCs for principal components

When run in its entirety, the *metaboprep* package performs six main actions: (i) extracts and processes (un)targeted metabolite data from source files, saving datasets in a standard tab-delimited format for use elsewhere; (ii) performs, when necessary, median normalization across (platform) batches; (iii) generates summary statistics from the initial raw dataset which are exported to a standard tab-delimited text file; (iv) performs sample and metabolite filtering according to user-defined thresholds and using a standard pipeline; (v) repeats the generation of summary statistics but on the filtered dataset and (vi) finally summarizes the data in an HTML report whilst also reporting on the influence of available batch variables. An overview of the workflow is shown in Figure 1 and a brief description given below. A log file is generated detailing each step taken in the pipeline including the filtering thresholds defined by the user and the number of samples or metabolites excluded at each step.

### 2.3 Running the R package

The package can be run via the command line using a parameter file but can also be run in an interactive mode using the functions built within. The parameter file contains key information for running the pipeline including the project name, the path to the source data directory, the input file names, the platform the metabolomics data is derived from and the preferred filtering thresholds. Thresholds and parameters to be provided include: (i) the fraction of metabolite missingness retained, (ii) the fraction of sample missingness retained, (iii) the total sum abundance (TSA) threshold in standard deviations from the mean, (iv) the outlier value threshold in interquartile range unit distance from the median for each metabolite, (v) a character indicator on how to treat outliers in the principal component (PC) analyses, (vi) a hierarchical clustering dendrogram tree cutting height in absolute Spearman’s rho units for data reduction prior to generation of PCs, (vii) the PC threshold for identifying outlying samples in standard deviations from the mean, (viii) a binary character declaring if derived variables should be excluded (TRUE or FALSE) and (ix) a character declaring a column name that should be used to perform batch normalizations.

### 2.4 Data extraction

Input data files can be in one of two possible formats, (i) excel spreadsheets as supplied by Nightingale Health or Metabolon; (ii) appropriately formatted tab-delimited text files (examples of these can be found on the GitHub repository). When an excel spreadsheet is provided as the source data, the package extracts (a) the (semi-)quantified metabolite data, (b) the associated sample metadata (e.g. technical batch information, sample identifiers) and (c) the associated metabolite metadata (e.g. metabolite class/pathway, HMDB identifier) and writes each ‘raw’ (i.e. unaltered) dataset to its own tab-delimited text file. Alternatively, the user can provide their (a) metabolite data, (b) optional sample metadata and (c) optional metabolite metadata, as appropriately formatted text files (.csv or .txt). The text files can contain metabolite abundance data from any platform. If samples are run in batches, this information can be provided in the preformatted text files or in commercial excel spreadsheets. Special considerations are made for certain metabolites when the data are derived from the commercial platforms of Metabolon or Nightingale Health. In the case of Metabolon data, metabolites labeled as ‘xenobiotics’ are excluded from calculations relating to sample missingness and do not follow the same processing pipeline as other metabolites. In the case of Nightingale Health data, many of the data summary and filtering steps are carried out having (optionally) excluded derived measures, which include several measures expressed as percentages or ratios.

### 2.5 Data summary

A data summary is generated twice by the package. Once on the raw, unaltered dataset or where necessary on the normalized dataset and again on the filtered (analysis ready) dataset. The summary includes a series of sample- and metabolite-based summary statistics. We use ‘sample’ here as a generic term that in many studies will mean the same as participant, as analyses to generate metabolite data will have been run on one sample per participant. However, as some studies will have repeat samples drawn over time from the same participants, sample, as used here means each individual sample on which metabolites are measured. Sample-based summary statistics include (i) an estimate of sample missingness, calculated as the proportion of missing (‘NA’) data and (ii) TSA [often referred to as total peak area (TPA) for those familiar with MS data], calculated for each sample by summing standardized (*z*-transformed and recentred to the absolute value of the datasets minimum) values across all metabolites. Metabolite TSA provides an estimate of the total (measured) metabolite concentration in the sample. Sample missingness is estimated using (a) all metabolites and again (b) to the exclusion of any defined list of metabolites, such as xenobiotics or derived measures. Sample TSA is estimated using (a) all metabolites and (b) again using only those metabolites with no missing data. Additionally, an outlier occurrence count, or an integer count of the total number of times an individual’s metabolite value is more than five interquartile range unit distances from the median metabolite concentration, is calculated and provided in the sample-based summary statistics file. Finally, subsequent to the estimation of metabolite summary statistics and the identification of representative metabolites, sample PCs are estimated, and the top 10 PCs provided in the summary data. This latter step is detailed below. Metabolite-based summary statistics include metabolite missingness, sample size (*n*) or the count of individuals without missing (‘NA’) data, and numerous other descriptive statistics including mean, standard deviation, skew and the coefficient of variance. A direct measure of each metabolites’ data distribution conformity to normality is provided by an estimate of Shapiro’s *W*-statistic, provided for both untransformed and log10 transformed data distributions. In addition, for each metabolite, a count of the number of outlying samples is provided as a further indication of skewness.

For the purposes of defining correlation structure and identifying a subset of approximately independent or ‘representative’ metabolites from the complete set of metabolites, a data reduction step is performed. These analyses provide users with a count of the effective number of metabolites in their dataset, which could be used for multiple testing correction, as well as a list of ‘representative’ metabolites. In the case of Nightingale Health NMR data or other data containing ratios of metabolites, derived measures can be excluded from this step. Further, data are restricted to common metabolites such that only those that are (i) variable and (ii) have less than or equal to 20% missingness are included. A dendrogram is then constructed [‘stats’ package hclust() function, with method ‘complete’] based on a Spearman’s rho distance matrix (1 − |Spearman’s rho|). A set of ‘*k*’ clusters (groups of similar metabolites) are identified based on a user-defined tree cut height (default 0.5 and equivalent to a Spearman’s rho of 0.5), using the function cutree() from the ‘stats’ package. For each ‘*k*’ cluster, the metabolite with the least missingness is then tagged as the representative metabolite for that cluster. Representative metabolites are identified by 1’s in the metabolite summary statistics file in the column ‘independent_features_binary’.

A principal component analysis (PCA) is conducted to evaluate interindividual variability in metabolomic profiles. This sample-based analysis uses only the reduced set of approximately independent ‘representative’ metabolites, as identified in the correlation analysis described above. Strictly for the purposes of deriving the PCs, missing values are imputed to the median and data then standardized (*z*-transformed) so that the mean equals zero and the standard deviation equals one for each metabolite. The variance explained for each PC is extracted and an estimate of the number of PCs (*n*) to retain is estimated, by both the acceleration factor and parallel analysis with the function nScree() from the ‘nFactors’ R package. The estimate of *n* derived by the acceleration factor, with a defined minimum of two, is used to identify sample outliers, i.e. those that deviate too far from the mean on those *n* PCs. By default, the outlier threshold is defined as five standard deviations from the mean, but this can be set by the user. In addition, if there is a concern that a few outlying metabolite values for a sample are causing that sample to be a PC outlier, users can choose—for the purposes of the PCA—to rerun the analysis with outliers converted to NAs or winsorized. Choosing the former option will result in the subsequent imputation of these values to the median (along with other missing values, as described above).

The summary statistics described above are written to two tab-delimited text files, one for samples and one for metabolites, and additionally once for the raw dataset and once for the filtered dataset. In addition, key statistics are reported (including graphically) in an HTML report (see below).

### 2.6 Data filtering

The next step in the pipeline is to derive a version of the metabolomics dataset which has undergone sample and metabolite filtering according to the user specifications provided in the parameter file (flow diagram in Supplementary Fig. S1). The first step is to remove, first samples, and then metabolites with extremely high rates of missingness (≥80%). In the second step, missingness is recalculated and sample and metabolite exclusions are made according to the user-defined thresholds for missingness with a default suggested value of 0.2 or 20%. Sample exclusions are then made on TSA, using only metabolites with complete data, according to user-defined thresholds with a suggested default of five standard deviations from the mean. Then, using all remaining samples and metabolites, the clustering and sample PCA steps described previously (as part of the data summary) are repeated. Results of the PCA are used to identify sample outliers for exclusion, defined as those that lie more than the user-defined threshold from the mean on *n* PCs where *n* is defined by the acceleration factor (as described previously). The default suggested threshold value is five standard deviations from the mean. This post-filtering version of the metabolite data is then passed back through the data summary procedure described above and finally exported in flat text format.

The most appropriate thresholds for sample and feature missingness will depend on both the total sample size and the intended analysis and we recommend users carefully consider the thresholds they set. Additionally, sample metabolome profile exclusions (TSA and PCA) are set at five standard deviations from the mean, as we have observed this to be a reasonable threshold to exclude samples that perform poorly, when sampling a random, presumptively healthy population. If sampling something like a case–control study design where extremes are perhaps expected or indeed a study sample with known substructure (e.g. different sample types) it would be advisable to evaluate the distributions presented in the HTML report (see below) and consider modifying these parameters.

### 2.7 HTML report

The standardized HTML report (designed for inclusion in papers in order to facilitate data description and hence transparency) includes the project name, the platform, a workflow image, data summaries and analysis of batch effects on key properties of the data—missingness and TSA. The data summary includes (1) an overview of the raw dataset: (1a) a visual of missingness in the data matrix, (1b) samples and metabolite missingness distributions; (2) an overview of the filtering steps: (2a) an exclusion summary, (2b) metabolite data reduction summary and (2c) a PC plot illustrating sample structure and identifying potential sample outliers; (3) a summary of the filtered dataset: (3a) count of remaining samples and metabolites, (3b) distributions for sample missingness, metabolite missingness and TSA, (3c) a metabolite clustering dendrogram highlighting the representative metabolites, (3d) a metabolite data reduction summary, (3e) a PC plot of sample structure, (3f) histograms for Shapiro *W*-statistic estimates across untransformed and log10 transformed metabolite abundances and (3g) a summary of sample and metabolite outliers. The report also includes an evaluation of the relationship between key sample properties (missingness rates and TSA) and potential batch variables, as provided by the user. Such variables might include sample storage box identifier, run day, super- and subpathway, sampling data and time and MS run mode. How these batch variables associate with missingness and TSA is illustrated in a series of boxplots that include an estimate of the variance explained by the batch, derived from a univariate analysis of variance and estimation of eta-squared using sums of squares. In addition, all the identified batch variables are placed in a type II multivariate analysis of variance and again the variance explained by each is summarized by the eta-squared statistic.

A power analysis for continuous and binary traits is provided based on the sample size of the dataset and using functions from the ‘pwr’ R package. If researchers are interested in the relationship between metabolites and a continuous trait (e.g. weight), power estimates are provided assuming a general linear model, whereas for the case of binary analyses (e.g. case/control) calculations are based on a two-sample *t*-test (allowing unequal sample sizes). The aim of these power calculations is to demonstrate the loss of power that can be expected because of varying degrees of missing data (i.e. as actual sample size decreases). Finally, the last step of the *metaboprep* pipeline is to write a PDF that contains, for each metabolite, a scatter plot identifying outlying data points and a histogram of the same values and a table that includes selected summary statistics. Together, the HTML and the PDF provide a quick overview and reference for the dataset.

### 2.8 Example datasets

#### 2.8.1 Born in Bradford—MS

The Born in Bradford (BiB; https://borninbradford.nhs.uk/) study is a population-based prospective birth cohort based in Bradford, United Kingdom. Full details of study methodology have been reported previously (Wright *et al.*, 2013). Ethical approval for the study was granted by the Bradford National Health Service Research Ethics Committee (ref 06/Q1202/48), and all participants gave written informed consent. For the data used in this example, women of White British (*N* = 500) or Pakistani (*N* = 500) ancestry were selected to have samples analyzed on the basis of their having complete data on a set of prespecified variables, including valid pregnancy fasting and post-load glucose measures, and both them and their index child having genome-wide data available [as described previously (Taylor *et al.*, 2021) and in Supplementary Fig. S2]. Samples were collected during pregnancy at around 24–28 weeks’ gestation. Participant characteristics are shown in Supplementary Table S1.

The untargeted metabolomics analysis of over 1000 metabolites was performed on these samples at Metabolon, Inc. (Durham, North Carolina, USA) on a platform consisting of four-independent UPLC-MS/MS runs. Detailed descriptions of the platform can be found in Supplementary Methods and in published work (DeHaven *et al.*, 2010; Evans *et al.*, 2009; Taylor *et al.*, 2021). The resulting datasets comprised a total of 1369 metabolites, of which 1000 were of known identity (named biochemicals) at the time of analysis. This dataset will be referred to throughout as BiB_MS-1.

#### 2.8.2 Avon longitudinal Study of Parents and Children—NMR

The Avon Longitudinal Study of Parents and Children (ALSPAC; http://www.bristol.ac.uk/alspac/) is a prospective birth cohort study, based in the former region of Avon, United Kingdom. Detailed information about the methods and procedures of ALSPAC can be found in Supplementary Methods and in published work (Boyd *et al.*, 2013; Fraser *et al.*, 2013; Northstone *et al.*, 2019). Ethical approval for the study was obtained from the ALSPAC Ethics and Law Committee and the Local Research Ethics Committees (a full list of all ethical approvals relating to ALSPAC are available online: http://www.bristol.ac.uk/alspac/researchers/research-ethics/). Specifically ethical approval for the clinic in which samples were collected for this work was granted by the National Research Ethics Service Committee South West—Frenchay (14/SW/1173). Consent for biological samples has been collected in accordance with the Human Tissue Act (2004).

NMR-derived metabolomics data were derived for 3361 EDTA-plasma/serum samples collected from 3277 unique individuals during the age 24 years clinic visit. Participant characteristics are shown in Supplementary Table S2. Quantification of selected circulating lipids, fatty acids and metabolites was performed using a 1D proton (^1^H) NMR spectroscopy-based platform from Nightingale Health (Helsinki, Finland). Spectra were acquired using standardized parameters using two NMR experiments or ‘molecular windows’ to characterize lipoproteins, low molecular weight metabolites and lipids. Further information relating to the data derivation can be found in Supplementary Methods and has been described previously (Inouye *et al.*, 2010; Soininen *et al.*, 2009, 2015). Raw metabolomics data preprocessing and quantification were as previously described (Inouye *et al.*, 2010; Soininen *et al.*, 2009, 2015). The resulting dataset comprised a total of 225 metabolites (including 78 derived measures); this dataset will be referred to throughout as ALSPAC_F24.

## 3 Results

**Fig. 2.**
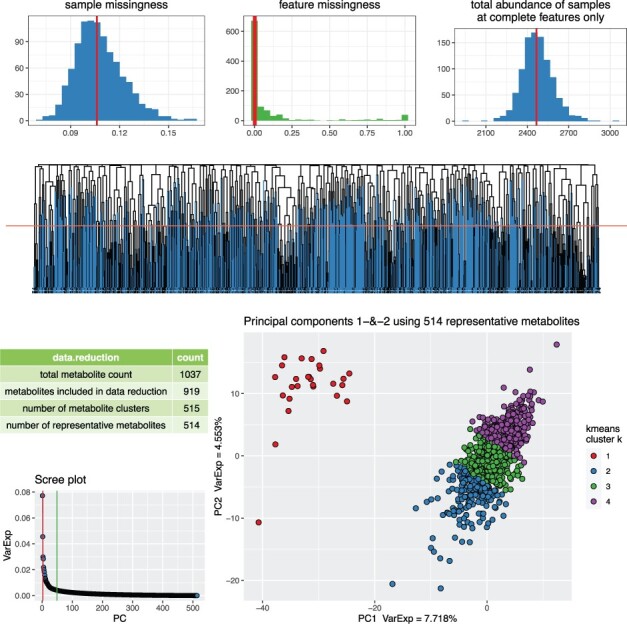
Summary figure found in each HTML report for the filtered dataset. There are seven figures in this BiB dataset summary figure. (1) The distribution of sample missingness. (2) The distribution for feature missingness. (3) The distribution for TSA, at complete features only. (4) A hierarchical clustering dendrogram based on absolute Spearman rho distances (1-rho) and cut at a tree cut height (red horizontal line) defined by the user. Blue branches on the dendrogram denote the features specified as ‘representative’ features used in the PCA. (5) A table of the number of metabolites used at each step of the dendrogram and PCA. (6) A scree plot of the variance explained for each PC also identifying the number PCs estimated to be informative (vertical lines) by the Cattel’s Scree Test acceleration factor (red, *n* = 2) and Parallel Analysis (green, *n* = 49). (7) A PC plot of the top two PCs for each sample. The number of metabolites used in the analysis is again indicated in the title of the PC plot. Individuals in the PC plot were clustered into four *k*-means (*k*) clusters, using data from the top two PCs. The *k*-means clustering and colour coding is strictly there to help provide some visualization of the major axes of variation in the sample population(s)

We used data from two established population-based cohorts (BiB_MS-1 and ALSPAC_F24) and two different analytical platforms (Metabolon and Nightingale Health) to demonstrate the utility of *metaboprep*. The summary HTML reports generated for each dataset can be found as Supplementary Data S2 and S3, respectively. The single core machine run times for the datasets were 3 and 10 min for ALSPAC_F24 and BiB_MS-1, respectively. An overview of each dataset based on the summary statistics generated by *metaboprep* prior to filtering are shown in Table 1. The choice of user-defined thresholds used in our analyses and the resulting exclusions made are summarized in Table 2. Based on the thresholds used here, 15 and 3 samples were excluded from BiB_MS-1 and ALSPAC_F24, respectively. No metabolites were excluded from ALSPAC_F24 whilst metabolite missingness criteria resulted in 24% of metabolites being excluded from BiB_MS-1. An example summary figure of the filtered data, which can be found in each HTML report, can be seen in Figure 2. Here, the BiB Metabolon dataset illustrates unexpected and pronounced sample substructure made obvious by the metaboprep steps (Fig. 2 and Section 4).

**Table 1.** Summary statistics for the initial, raw (prefiltered) BiB_MS-1 and ALSPAC_F24 datasets

	BiB_MS-1	ALSPAC_F24
**Summary statistic**		
Platform	Metabolon	Nightingale Health
No. of samples	1000	3361
No. of metabolites	1369	225
**Sample statistics**		
% sample missingness^a^ (min, median, max)	11.85, 18.45, 26.81	0.00, 0.00, 12.24
TSA at complete metabolites (min, median, max)	1.85, 2.35, 2.98 (×10^3^)	3.99, 4.31, 4.75 (×10^3^)
Count of outlying data points per sample (min, median, max)	0, 5, 105	0, 0, 48
**Metabolite statistics**		
% metabolite missingness (min, median, max)	0, 2.6, 100	0.00, 0.00, 1.71
Count of outlying data points per metabolite (min, median, max)	0, 2, 99	0, 2, 344
% with *W*-statistic ≥0.95	15.49	42.22
% whose *W*-statistic decreases following log_10_ transformation	9.2	44.89
No. of representative metabolites	512	24

*Note*: The table provides details on the platform, sample size, sample and metabolite missingness, TSA for samples, and outlier counts, the percent of metabolites that may be considered normal distributed and an estimate of the number of representative metabolites in the dataset.

a Calculated after the exclusion of derived variables in the Nightingale Health dataset and of xenobiotics in the Metabolon dataset.

**Table 2.** Results of sample and metabolite filtering based on default exclusion thresholds

Filtering step	Exclusion threshold	BiB_MS-1	ALSPAC_F24
Raw dataset (prefiltering)		1000 samples	3361 samples
		1369 metabolites	225 metabolites
1. Extreme sample missingness^a^	≥80%	0	0
2. Extreme metabolite missingness^a^	≥80%	96	0
3. Sample missingness^a^^,^^b^	≥20%	3	0
4. Metabolite missingness^a^^,^^b^	≥20%	236	0
5. Sample TSA^b^^,^^c^	>5SD	1	3
7. PCA outliers^b^^,^^d^^,^^e^	>5SD	11	0
Final dataset (post-filtering)		985 samples	3358 samples
		1037 metabolites	225 metabolites

*Note*: PCA, principal component analysis; SD, standard deviations.

a Calculated after excluding metabolites in the xenobiotic class from Metabolon data and derived measures from Nightingale Health data.

b User-defined threshold. Rows in blue are sample filtering steps.

c Derived from complete metabolites only.

d Excluding metabolites with >20% missingness.

e Using the representative metabolites only and excluding on the number of PCs determined by the acceleration factor with a minimum of two PCs.

## 4 Discussion

In this paper, we have presented *metaboprep*, an R package for use by researchers working with curated, high quality, metabolomics data and developed in the context of population health research. The package enables metabolomics data from different platforms to be extracted, processed, summarized and prepared for subsequent statistical analysis within a standardized and reproducible workflow. This work was motivated by the need for increased consistency and transparency in the preanalytical processing of data across cohorts and studies, but also acknowledges that a ‘one size fits all’ approach is unlikely to be feasible given the range of study designs being employed. Metabolomics is a growing field within population sciences, with application to a vast array of hypotheses. As such, research groups have differing approaches to data preparation, which can make results hard to interpret and compare across studies. It is important to understand the properties of metabolomics data in order that suitable preanalytical processing steps can be performed, and downstream analytical results interpreted appropriately. When combined with the ongoing efforts of individuals (Zhang *et al.*, 2020) and organizations such as the Metabolomics Quality Assurance and Quality Control Consortium (mQACC; Beger *et al.*, 2019; Evans *et al.*, 2020) to improve the quality of data being delivered to researchers by laboratories, we hope the use of transparent processing pipelines, such as *metaboprep*, will drive up the quality and transparency of metabolomics research in epidemiology.

In the proposed pipeline, considerations were made for two specific conditions within two platforms currently available. It is difficult to mitigate against future developments, but the *metaboprep* approach is able to accommodate specific flags as they appear. In this case, xenobiotics—common in the Metabolon dataset, and derived variables—common in the Nightingale Health dataset. Xenobiotics are exogenous metabolites (i.e. not produced by the body), such as drug compounds and a quantified measure indicates presence of the exogenous compound. Consequently, they can have very high rates of missingness, whilst still being critically informative to a study as the majority with missing data will be a true ‘no’ for exposure to the exogenous compound. For this reason, we do not exclude xenobiotics on the basis of high missingness but would advocate affording them special consideration in any downstream statistical analyses. For example, these metabolites might best be evaluated within a presence/absence framework rather than by analysis of relative abundance. In data from Nightingale Health, derived variables are metabolite traits that are a summary of two or more other metabolites (possibly already represented in the dataset) or ratios of two or more metabolites. These variables can introduce bias in estimates of sample missingness (where a single metabolite is missing, any derived measures based on that metabolite will also be missing) and may not be appropriate to retain when identifying a set of representative metabolites for the dataset. We allow the user to include or exclude the derived variables in the pipeline at their discretion.

One of the most commonly implemented preanalytical steps is filtering based on missingness. Missingness is defined as the proportion of data with no value and can vary hugely within a dataset (0–99.9% missing). Typically, researchers filter metabolites on missingness to remove metabolites that exhibit evidence of technical error, or where the proportion of missingness introduces downstream analytical difficulties. Conversely, the filtering of samples based on missingness helps identify samples that may have been of poor quality or mishandled before or during the metabolomic assay(s). However, without external data the nature of the missingness, and the extent to which removal of samples or metabolites introduces more or less bias than not excluding these (and possibly imputing missing data) is unclear (Hughes *et al.*, 2019) and will vary by sample size and the intended main research questions. Deciding upon appropriate missingness thresholds can be critical to a study and some caution and consideration are warranted. This results from the variety of reasons for missing data in this context—e.g. the technology, signal-to-noise ratios, signal intensity, error (Do *et al.*, 2018; for further discussion on missingness see Supplementary Discussion). Crucially, missingness might also represent true absence and thus be informative for some biological hypotheses, e.g. differential missingness by class (e.g. case/control status or sex). For this reason, our workflow allows uses to define the thresholds they want to apply for missing samples and metabolites, the thresholds for these two can be different and either or both can be zero (no exclusions based on missing data). This allows researchers to repeat the workflow with different thresholds to explore the extent that these influence main analysis results.

TSA is a sample-based metric estimated for the purposes of identifying samples with broad quality issues, such as handling errors (i.e. differing concentrations of sample) and is calculated by summing values across all metabolites. This metric is, by definition, correlated with missingness rates, so is estimated a second time here using only complete metabolites with this latter metric being used in the exclusion step. In order to guard against selection bias, the implementation of this exclusion step should be considered carefully and within the context of the study design. There may be situations whereby a high (or low) TSA is indicative of a true biological state, rather than of any technical issue. For example, if the coverage of the metabolomics platform is skewed towards a class of metabolite, e.g. lipids, then certain characteristics of individuals in the study sample may be correlated with the TSA measure, e.g. body fat percentage. Alternatively, if a study design were to include data from various tissues, then the TSA distribution may be bimodal and basing exclusions on standard deviations from the mean may be difficult if not inappropriate. For these reasons, the TSA distribution is provided in the HTML report for assessment by the user who may then choose to explore the sensitivity of downstream analyses to the application of different thresholds.

The proposed workflow provides information relating to structure within the study sample. This is done by implementation of sample-based PCA with summary data provided in the summary statistic files and corresponding plots for visual inspection. Only metabolites with limited missingness (<20%) are included in these analyses to avoid the need to implement a probabilistic PCA whilst limiting the introduction of error by the simplified (median-based) imputation—an imputation used strictly for deriving PCs. Furthermore, data reduction to remove highly correlated metabolites is considered necessary to ensure that the estimated PCs are not driven by any common, highly correlated metabolite classes, pathways or clusters. Taking this approach, the PCs should provide an equally represented, broad perspective of variation in the data. If a sample is mishandled, the assumption would be that all assayed variables would be perturbed, and this would be evident in the PCA. However, it is possible that a few extreme values can push a sample to be an outlier in PC analyses. As such, users can have outlying values turned into NAs or winsorized to the maximum of all remaining values prior to the PCA to insure they are not removing a sample because of a few errant observations. Outlying values that are optionally turned into NAs or winsorized are not an output of *metaboprep*, they are merely internal steps taken for the estimation of PCs. Just as discussed with missingness and TSA metrics, proper consideration for thresholds is important here too. Outliers may be biologically relevant and gross structure may be present if multiple tissues, populations or species are sampled. If that is the case, just as for TSA, then thresholding on standard deviations from the mean may be a difficult if not inappropriate filtering step. However, if you are anticipating a homogenous sample but observe clustering (as in Fig. 2 PC plot), then you should attempt to identify the source of the clustering and potentially reconsider your PCA sample filtering threshold.

Three preprocessing steps that the *metaboprep* pipeline does not, currently, incorporate is modification of outlying data points (winsorization or truncation), data transformation and imputation. Each of these topics bring with them their own particular issues and considerations that are beyond the scope of the current package. We will however note that whilst log transformations appear to be commonly applied to metabolomics datasets—64% of COMETS (The Consortium of METabolomics Studies) responding cohorts claim to routinely log transform their data (Playdon *et al.*, 2019)—we routinely observe that this does not always generate an approximate normal distribution and at times can make data distributions less normal. Shapiro *W*-statistics (a metric for normality) are provided alongside outlier flags in the summary statistic file for metabolites and the distribution of *W*-statistics for the raw and log-transformed data is provided in the PDF report. We encourage use of this information to aid decisions regarding the most appropriate data transformation(s), given the intended statistical analyses. These considerations should also include the research question, including whether the metabolites are exposures or outcomes, and the planned main analyses.

To date, the *metaboprep* package has only been used to process ^1^H NMR and LC-MS metabolomics data and Olink (proximity extension assays) proteomics data derived from serum or plasma. Whilst we do not anticipate any issues in processing data derived from other platforms (GC-MS) or sources (urine, tissue), users should consider carefully whether the assumptions we make are appropriate in these scenarios. The same is true if the package is used for processing small samples (*n* < 20), where steps such as estimating means, medians, correlation coefficients and data quality metrics may not perform optimally due to decreased precision. We reiterate that the workflow presented here does have its compromises. As highlighted above, data preparation does not end with the running of this workflow but with the careful evaluation of the data reports provided by it. Going forward, *metaboprep* will be developed to address evolving needs, starting with additional functionality to enable direct read-in of the new (since 2021) format datafiles supplied by Metabolon. Perhaps unsurprisingly, given the rapid increase in use of metabolomics data in epidemiology, parallel efforts are being made to improve analytical efficiency, such as the recent release of the R package maplet (Metabolomics Analysis PipeLinE Toolbox; Chetnik *et al.*, 2022), and to construct pipelines for combining metabolomic datasets across cohorts (Viallon *et al.*, 2021); any future developments of *metaboprep* will necessarily be made within this context. Our package does not provide any tools for statistical analysis or downstream interpretation, and therefore, we anticipate that *metaboprep* will be used in conjunction with complementary tools such as MetaboAnalyst (Pang *et al.*, 2021), which provides a broader set of functions to aid raw MS spectra processing as well as post-analytical biomarker analysis.

In conclusion, in the interests of open science and to encourage collaboration, we present a first release of *metaboprep*, an R package that we hope to develop further in response to feedback from the community. In this paper, we have avoided making definitive recommendations regarding thresholds that should be used since these should be chosen in the context of the specific study design and research question. We encourage those working with curated metabolomics data to use our package to enhance their understanding of the characteristics of their metabolomics data, its structure and how these properties could impact on downstream statistical analyses and importantly, to report their findings alongside the results of their main analyses.

## Supplementary Material

btac059_Supplementary_Data
